# Developmental Neuropathology and Neurodegeneration of Down Syndrome: Current Knowledge in Humans

**DOI:** 10.3389/fcell.2022.877711

**Published:** 2022-05-23

**Authors:** Zinnat Hasina, Nicole Wang, Chi Chiu Wang

**Affiliations:** ^1^ Department of Obstetrics & Gynaecology, The Chinese University of Hong Kong, Shatin, Hong Kong SAR, China; ^2^ School of Veterinary Medicine, Glasgow University, Glasgow, United Kingdom; ^3^ Li Ka Shing Institute of Health Sciences, School of Biomedical Sciences, Chinese University of Hong Kong -Sichuan University Joint Laboratory in Reproductive Medicine, The Chinese University of Hong Kong, Shatin, Hong Kong SAR, China

**Keywords:** Down syndrome, brain development, neuropathology, neurodegeneration, human

## Abstract

Individuals with Down syndrome (DS) suffer from developmental delay, intellectual disability, and an early-onset of neurodegeneration, Alzheimer’s-like disease, or precocious dementia due to an extra chromosome 21. Studying the changes in anatomical, cellular, and molecular levels involved may help to understand the pathogenesis and develop target treatments, not just medical, but also surgical, cell and gene therapy, etc., for individuals with DS. Here we aim to identify key neurodevelopmental manifestations, locate knowledge gaps, and try to build molecular networks to better understand the mechanisms and clinical importance. We summarize current information about the neuropathology and neurodegeneration of the brain from conception to adulthood of foetuses and individuals with DS at anatomical, cellular, and molecular levels in humans. Understanding the alterations and characteristics of developing Down syndrome will help target treatment to improve the clinical outcomes. Early targeted intervention/therapy for the manifestations associated with DS in either the prenatal or postnatal period may be useful to rescue the neuropathology and neurodegeneration in DS.

## Introduction

### Neuropathology and Neurodegeneration in Down Syndrome

Down syndrome (DS) is a genetic disorder caused by a third copy of chromosome 21 (Chr21) instead of the original two (Cenini et al., 2012). This extra Chr21 gives rise to many congenital abnormalities of DS during prenatal and postnatal development (Dierssen et al., 2009). Many affected infants develop various extents of clinical manifestations like infantile spasms, epileptic seizures, intellectual disability, and precocious dementia (Malt et al., 2013). Nowadays, the life expectancy of individuals with DS is increasing more than ever due to the vast advancement of more sophisticated scientific technology and medical procedures. Individuals with DS live healthier and longer, but with an intellectual disability. Intellectual disability (ID) is a common neurological deficit in DS; almost all individuals with DS have mild to moderate ID (Krasuski et al., 2002). Any disruption during the development of the nervous system refers to neuropathogenesis. Until now, the underlying cause of the ID in DS was not clearly known; it may be due to altered anatomical structure, cellular functions, and molecular pathways resulting in dysfunction of higher brain functions (Rachidi and Lopes, 2010; Valenti et al., 2014). Previous research has been conducted more on animal models of DS than on individuals with DS, and also more on the postnatal brain than on the prenatal brain. The observations made in the studies performed in the mouse models of DS may not be comparable and replicable in humans. In addition, individuals with DS are prone to developing an early onset of dementia or Alzheimer’s disease (precocious dementia). Dementia is a gradual loss of cognitive functions (learning, thinking, attention, decision making, and problem-solving) (Fisher et al., 2019) that hampers daily life. These neuropathological changes in the brains of individuals with DS may start from the early developmental stage, as trisomy 21 is the result of chromosomal nondisjunction during meiosis, before fertilization (Dierssen et al., 2009).

Human brain development occurs in the 3rd week of pregnancy and is morphologically completed at birth as an immature form of the adult brain, though neurogenesis carries on up to adulthood (Stiles, 2008; Duane and Gregory, 2018a). The early postnatal period is also a valuable time for brain development. Interactions of basic physical, social, emotional, intellectual, language, and self-helping skills generate the infrastructure for future neuro-social development (Stiles, 2008; ^1^). Manifestations in the early developmental stage are important in recognizing the underlying neuropathological mechanisms responsible for the DS phenotypes. Here we described the neuropathogenesis of the human brain from conception to adulthood of individuals with DS at anatomical, cellular, and molecular levels, finding gaps in the field, building molecular networks within the genes of chromosome 21 and also between non-chromosome 21 and chromosome 21 genes for better understanding the mechanism and clinical importance of this analysis/review. Studying the changes in anatomical, cellular, and molecular levels involved may help to understand the pathogenesis and develop target treatments, not just medical, but also surgical, cell and gene therapy, etc., for individuals with DS. In this review, we summarized only human data. A review of animal data for neuropathogenesis in DS has been published in another review already (Haydar and Reeves, 2011).

## Methods

### Sources and Criteria

We performed our literature search through interrogation of citations from PubMed, MEDLINE, and EMBASE for the past 30 years. The keywords included Down syndrome, brain development, neuropathology, neurodegeneration, and human. Additional potential papers were searched from the reference lists of the searched papers. GeneCards and NCBI (National Center for Biotechnology Information) were used for gene information. All published literature of cohort studies for prenatal and postnatal DS in brain development and pathological studies were included. We selected papers that included their participants confirmed as trisomy 21 and used human brain tissues or cells regardless of imaging, autopsy, or histopathology for their study specimen. Animal studies, cell line studies, and specimens other than brain tissues or cells were excluded. Debate and commentary papers, imaging and laboratory studies for screening and review papers were also excluded from our review.

### Data Extraction and Synthesis

Initially, we identified 1,568 (164 embryonic brain, 419 foetal brain, and 985 postnatal brain development in DS) articles for anatomical and cellular studies, and 615 (509 Chr21 & 106 non-Chr21) articles for our molecular studies from the literature search. At the end, 16 eligible anatomical and cellular studies were selected and 17 (Chr21) and 7 (non-Chr21) eligible molecular studies were included for data extraction. Demographic data (including developmental age/age at necropsy), controls, sample size, brain parts, outcomes measures (including markers and methods), and results (anatomical, cellular, and molecular changes in the whole brain and different parts of the brain) from the selected articles were collected. We recorded both positive and negative results, fold changes of gene intensity, mean and standard deviation (SD), or percentage of the incidence, and *p* values of the outcomes.

## Brain Development at the Anatomical Level

The human brain is a complex organ made up of well-organized segments that perform jointly to control the functions of the body and to regulate the higher functions of the mind that make us human (Mark et al., 2005; Duane and Gregory, 2018d). The development of the human brain starts as a neural groove from a distinct part of the ectoderm, the neuroectoderm, and forms a neural tube after the end of the 3rd week of gestation (Bronner-Fraser, 2002; Stiles, 2008; Tau and Peterson, 2010). The primary and secondary brain vesicles appear through progressive production of neural tissue from the neural tube around the 4^th^ and 5^th^ weeks of gestation, respectively (Duane and Gregory, 2018e; O'Rahilly and Müller, 1990). The brain ventricles create cerebrospinal fluid, which regulates the brain’s interstitial fluid homeostasis and acts as a hydromechanical protector of the central nervous system (O'Rahilly and Müller, 1990).

### Cerebrum

Cerebral hemispheres appear around the end of the 5^th^ week of gestation from lateral expansions of the telencephalon (Sadler, 2012; Duane and Gregory, 2018f). The brain cortex, composed of more than 100 billion neurons, develops from the 6th week of gestation and ends at birth (Stiles, 2008). There is a positive relationship between total brain volume (TBV) and intelligence quotient (IQ) (McDaniel, 2005). In 2 to 6-month-old infants with DS, brain weight decreases about 1.2-folds less than in similar-aged normal infants (Schmidt-Sidor et al., 1990). Total intracranial volume (TIV) was reduced 1.1-folds less in children and adolescents with DS (7–16 years old) than in similar aged normal controls (Carducci et al., 2013). TBV decreased 1.2-folds, total grey mater volume 1.2-folds, and total white mater volume 1.3-folds in 5–23 years old individuals with DS (Pinter et al., 2001). Reduction of cerebral cortex (1.1-folds) and white mater (1.2-folds) volume was seen in 30–45 years old individuals with DS (Weis et al., 1991). TBV & GM also decreased 1.3 & 1.4-folds in individuals with DS over 40 years old compared to normal controls (Pearlson et al., 1998). This suggests that brain volume reduction in DS is consistent with the aging process related to the early onset of dementia and Alzheimer’s disease in later life.

**FIGURE 1:**
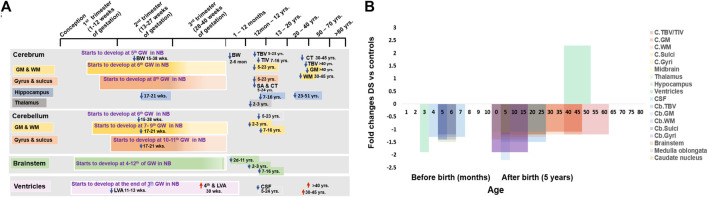
Anatomical changes in human Down syndrome brains. **(A)** Time line of major brain structures during development in normal (color boxes) and Down syndrome (DS, color arrows). **(B)** Fold changes of various brain structure volumes or sizes in DS vs. normal controls. Mean change data is extracted from available literature as shown in Table 1. NB = normal brain; DS = Down syndrome; ↑ = increase; ↓ = decrease; GW = gestational week; GM = Gray matter; WM = White matter; TBV = total brain volume; TIV = total intracranial volume; LVA = lateral ventricular area; CPA = choroid plexus area; CSF = cerebrospinal fluid; C = cerebral; Cb = cerebellar.

Sulci and gyri start to develop at the 8^th^ week of gestation and continue till birth. Sulci and gyri increase the surface area of the brain and, therefore, increase behavioral and intellectual ability (Duane and Gregory, 2018g; Stiles and Jernigan, 2010). While cortical thickness is negatively related to general intelligence in children and adolescents (9–16 years), it is positively related in younger adults (16–24 years) (Menary et al., 2013) (Figures 1A,B and Table 1). Cortical thickness increases 1.1-folds and total surface area decreases 1.1-folds in 5–24 years old individuals with DS (Lee et al., 2016). Superior temporal gyrus reduces 1.2-folds in 5–23 years old individuals with DS than in aged-matched controls (Pinter et al., 2001). This suggests that the brains of individuals with DS are comparatively smaller and with fewer superficial sulci and gyri than in normal individuals, resulting in a ‘Lissencephalic’ or smooth brain surface and contributing to general cognitive and developmental deficits (Duane and Gregory, 2018a) (Figure 1B and Table 1).

**TABLE 1:** Summary of neuropathogenesis at anatomical, cellular, and molecular levels in human.

Studies	Study design	Country	Sample size		Mean age/range		Brain parts	Methods	Markers	Anatomical changes	Cellular changes	Molecular changes	Implications	Limitations
			DS	Controls	DS	Controls								
Griffin et al. (1989)	Case control	United States	3	3	2 days to 3.5 months	2 days to 3.5 months	Temporal lobe	IHC	IL-1 & GFAP	-	-	↑IL-1	1 IL-1 may forecast early onset Dementia.	Very small sample size.
Schmidt-Sidor et al (1990)	Case control	United States & Poland	101	80	Birth to 60 months	Birth to 60 months	Whole brain	Gross neuropathological study	Brain weight (BW)	↓ BW.	-	-	↓ed BW may contribute MR, & DA.	Samples were of a wide age range.
Weis et al. (1991)	Case control	Austria	7	7	30-45 yrs.	36-44 yrs.	Whole brain	CT scan & MRI scan	T1 & T2-weighted images.	↓ Cerebral cortex, WM & ↑ ventricles		-	Volumetric structural brain differences were seen in adult DS.	Smaller study size
Kesslak et al. (1994)	Case control	United States	13	10	23-51 yrs.	23-51 yrs.	Whole brain	MRI scan	T1 & T2-weighted images.	↓ hippocampal & ↑ ventricular area	-	-	Enlarged ventricles may reflect neuropathologic changes at cellular level.	Small sample size & wide age range
Arai et al. (1995)	Case control	Japan	29	20	20 GW to 50 yrs.	20 GW to 50 yrs.	Frontal cortex	IHC	Apo-E IR astrocytes	-	-	Apo-E IR astrocytes ↓ in foetal brain, ↑ in adult brain.	Altered apo-E producing astrocytes may point to the early onset of dementia.	Samples were of a wide age range.
Pearlson et al. (1998)	Case control	United States	50	23	42.39 ± 10.45 yrs. (>40 yrs.)	42.39 ± 10.45 yrs. (>40 yrs.)	Whole brain	MRI scan	T1 & T2-weighted images.	↓TBV & GM, ↓ hippocampal volumes & ↑ ventricular size.		-	Generalised atrophy & ventricular hypertrophy.	Small number of participants of DS with dementia.
Griffin et al. (1998)	Case control	United Kingdom	20	16	17 GW to 68 yrs.	22 GW to 68 yrs.	Cerebrum	IHC	S100β+, GFAP & βAPP	-	-	↑ S100β+ astrocytes & β-APP expressing neurons.	Overexpression of S-100 may cause AD in DS.	Samples were of a wide age range.
van Leeuwen et al. (1998)	Case control	Netherlands	7	12	1. <65 yrs.	1. <65 yrs.	Cerebral cortex & hippocampus	IHC	Ubi-B+1 (Ubiquitin)	-	-	1. Ubi-B+1.absent	Ubi-B+1 in the NFT and dystrophic neurites may lead to AD in DS.	Age was not possible to match at death.
					2. >65 yrs.	2. >65 yrs.						2. ↑ Ubi-B+1 in DS		
Pucharcós (1999)	Case control	Spain	2	2	18-22 GW	18-22 GW	Prosencephalon	RT-PCR	ITSN1	-	-	↑ITSN1	↑ed ITSN may contribute to some of the abnormalities of DS.	Very small sample size.
Saito et al. (2000)	Case control	Japan	37	29	34 GW to 60 years	33 GW to 70 years.	Cerebrum & cerebellum	WB & IHC	DSCAM	-	-	Positive DSCAM IR in cerebral & cerebellar cortical neurons.	DSCAM may play a role in the myelination process during development.	Samples were of a wide age range.
Pinter et al. (2001)	Case control	United States	16	15	5-23 years	5-23 years	Whole brain	MRI scan	T1 & T2-weighted images.	↓ TBV, GM, WM, C, Cb & STG.	-	-	Overall smaller & smooth brain in DS.	Smaller study size & wide age range
Gulesserian et al. (2001a)	Case control	United Kingdom	9	9	56.1±7.1 years	72.6±9.6 years	Cerebral cortex	MALDI	SOD1	-	-	↑ SOD-1 levels.	↑ed SOD-1 levels may responsible for the oxidative stress.	Very small sample size.
Arai et al. (2002)	Case control	Japan	16	25	16 GW to 50 years	7 GW to 70 years	Frontal lobe	WB & IHC	SYNJ IR in neurophils or cytoplasms of CRC	-	-	↑ed SYNJ in CRC in foetal period & CP neurons throuout life.	↑ed SYNJ may disrupt the neuronal migration and synaptogenesis	Small sample size & wide age range.
Motonaga et al. (2002)	Case control	Japan	13 & 4	15	1. 27 GW to 32 yrs. (without dementia)	1. 27 GW to 32 yrs.	Frontal cortex	IHC	BACE2			1. BACE2 IR not detected	↑ed BACE2 may involves in AtD.	Samples were of a wide age range.
					2. 49-60 yrs. (with Dementia)	2. 49-60 yrs.						2. BACE2 IR detected in DS.		
Shim et al. (2003)	Case control	United Kingdom	6	6	57.8±8.2 years	60.2±9.3 years	Frontal cortex	WB	ERG, SIM2 & RUXN1	-	-	↑ ERG. ↑SIM2 & RUXN1 (NS)	↑ed apoptotic cell death & neurodegeneration.	Very small sample size.
Sánchez-Font et al. (2003)	Case control	Spain	12	10	18 - 23 GW	18 - 23 GW	Cerebrum	RT- PCR -northern blot hybridizations	FABP7 & PKNOX1			↑ PKNOX1 & ↑ FABP7	Transactivation of the FABP7 gene promoter is due to PKNOX1 overexpression	Smaller sample size.
Shin et al. (2004)	Case control	Spain	8	7	19.8±2 GW	18.8±2.2 GW	Cerebral cortex	MALDI	GATD3A / ES1	-	-	↑ ES1 level.	Overexpression of ES1 ↑ed transcriptional activity.	Very small sample size.
Ferrando-Miguel et al. (2004)	Case control	Spain	8	6	19.4 ± 1.1 GW	19.1 ± 1.6 GW	Frontal cortex	WB	DSCR5, DSCR6, DSCR4 & GIRK2 / KCNJ6	-	-	↑DSCR5 & DSCR6, & ↓ GIRK2 (NS).	DS phenotype explained not only by the gene dosage effect hypothesis.	Very small sample size.
Guihard-Costa et al. (2005)	Case control	France	355	922	15-38 GW	15-38 GW	Whole brain	Precision balance- precision of 1 g.	BW	↓ BW.	-	-	Restriction of brain growth in DS foetuses as early as 15 weeks	
Colombo et al. (2005)	Case control	United States & Argentina	12	15	3 months to 69 years	16 days to 69 years	Cerebrum	IHC	GFAP	-	↓ IGP composed by astroglial cells.	-	Alteration of astroglia may involves AtD during adult life.	Very small sample size.
Guidi et al (2008)	Case control	Italy	7	6	17- 21 GW	17- 21 GW	Hippocampal region	IHC	Ki-67, GFAP & NeuN	↓ hippocampus & ventricular zone.	↓ proliferative cells in the hippocampalaregions & ventricular zone.	-	↑ed apoptotic cell death reduced neurons number.	Very small study size.
Cheon et al. (2008)	Case control	Spain & United Kingdom	4 & 8	4 & 9	1. 18-19 GW	1. 18-19 GW	Cerebral cortex	WB	APP, BACE1 & BACE2	-	-	1. ↑ APP & BACE in DS (NS)	Disruption of APP function may cause neurodegeneration	Very small sample size.
					2. 55.88 ± 7.97 yrs.	2. 55.88 ± 7.97 yrs.						2. ↑APP in DS.		
Rachidi et al. (2009)	Case control	France	6	6	22 -24 GW	22 -24 GW	Cerebral cortex, hippocampus & cerebellum	Radioactive in situ hybridization and Quantitative expression analysis	C21orf5 mRNA	-	-	↑ C21orf5 & differential expression in hippocampus, cerebral & cerebellar cortex.	Plays a potential role in functional brain alterations	Very small sample size & small age range.
Guidi et al (2011)	Case control	Italy	7	6	17- 21 GW	17- 21 GW	Cerebellum	Anatomy of cerebellum & IHC	Ki-67	↓ Cerebellar fissure, lobuli, GM & WM volume.	↓ EGL & IGL	-	Generalized hypocellularity	Very small sample size.
Michael et al. (2011)	Case control	United States	25	16	5 months to 62 years	6 months to 67 years	Frontal cortex	WB	ITSN1-S	-	-	↑ ITSN1-S	↑ed ITSN1-S may disrupt normal functioning of neurons	Small sample size & wide age range.
Loureiro et al. (2012)	Case control	United Kingdom	63	410	11-13 GW	11-13 GW	Ventricle	TVUS	LVA in a transverse view	↓ LVA	-	-	↓ed LVA may be due to ↓ed brain volume.	Smaller patient size.
Dowjat et al. (2012)	Case control	United States	25	20	21 days to 65 years	14 days to 85 years	Frontal cortex	IHC and Confocal Microscopy	Anti-DYRK1A 8D9 and β-actin antibodies	-	-	↑ DYRK1A in newborns, infants & adults.	↑ed DYRK1A may cause MR & cognitive deficits.	Samples were of a wide age range.
Lu et al. (2012)	Case control	United States	7	7	14 - 18 GW	14 - 18 GW	Frontal cortex	WB	OLIG2, PCNA & KCNA3	-	-	↑ OLIG2 & ↓ KCNA3	↑ OLIG2 inhibits neural progenitor proliferation.	Very small sample size.
Carducci et al. (2013)	Case control	Italy	21	27	7-16 years	7-16 years	Whole brain	MRI scan	T1 & T2-weighted images.	↓ TIV, GM, WM, hippocampus, & brainstem	-	-	↓Volume observed throughout childhood, adolescence and adulthood.	Low resolution of MRI data.
Kanaumi et al. (2013)	Case control	Austria	28	30	14 GW to newborn	14 GW to newborn	Cerebrum	IHC	CD68, HLA-DR, Olig2 and TPPP/p25 and GFAP	-	Altered production of neuronal & non-neuronal cells.	-	May involves in defective neurogenesis.	Small sample size and wide age range.
Martin et al. (2014)	Case control	United States	39	28	1. ≤40 yrs.	1. ≤40 yrs.	Frontal cortex	WB	SYNJ1	-	-	1. ↑ SYNJ1	SYNJ1 overexpression is higher with AD in DS.	Age wasn’t possible to match at death.
					2. >40 yrs. (DSAD).	2. >40 yrs.						2. ↑ SYNJ1		
Perluigi et al. (2014)	Case control	United States	16	16	1. 23.3±16.8	1. 24.8±11.6 2. 57.2±7.6 yrs.	Frontal cortex	WB	RCAN1 / DSCR1	-	-	1. ↑ RCAN1	May involve in the formation of NFTs in DS, like as AD	Small sample size.
					2. 59.5±3.2 yrs.							2. ↑ RCAN1		
Lee et al. (2016)	Case control	United States	31	45	5 -24 years	5 -24 years	Whole brain	MRI scan	T1-weighted images	↑ CT, ↓ SA, GM & WM & CSF	-	-	↓ SA due to ↓ volume of GM & WM.	Wide age range samples.
Chu et al. (2016)	Case control	United States	3	3	55.8±1.7 years	64.6±1.9 years	Frontal cortex	WB & IHC	GSAP	-	-	↑ GSAP	GASP activates the Aβ formation.	Very small sample size.
Gunbey et al. (2017)	Case control	Turkey	10	8	2.6 ± 0.69 yrs. (2-3 years)	2.5 ± 0.707 yrs. (2-3 years)	Whole brain	MRI scan	TBSS for DTI	↓ Cerebellar GM & WM, thalamus, caudate nucleus & brainstem.	-	-	This structural alteration may reflect the neurodevelopmental delay.	Small patient group.
Fujii et al. (2017)	Case control	Japan	32	32	2 days to 11 years	9 days to 11 years	Brainstem	MRI scan	T1-weighted images	↓ brainstem, pons, midbrain, medulla oblongata	-	-	Smaller brainstem is due to congenital hypoplasia.	The position of the children at the MRI may affect measured values.
Tramutola et al. (2020)	Case control	Italy	6	8	<40 yrs. old	<40 yrs. old	Frontal cortex	WB	IRS1 & IR			↓IRS1 & IR (NS)	May neuropathology like AD in young people with DS	Small sample

DS, Down syndrome; MR, mental retardation; DA, developmental abnormalities; ↑ Increase; ↓ Decrease; GW, gestational week; USA, United States; UK, United Kingdom; IL-1, interleukin 1; GFAP, glial fibrillary acidic protein; MALDI, matrix-assisted laser desorption ionization; ApoE, apoprotein E; IR, immunoreactivity; RT-PCR, real time polymerase chain reaction); CD68, cluster of differentiation 68; HLA-DR, human leukocyte antigens DR isotype; Olig2, oligodendrocyte transcription factor; TPPP/p25, tubulin polymerization promoting protein; Ki-67, marker of proliferation; S100β+, S100 calcium-binding protein B; ITSN1-S, intersectin1; DSCAM, Down syndrome cell adhesion molecule; SOD1, superoxide dismutase 1; APP, amyloid precursor protein; βAPP, amyloid-beta precursor protein; NeuN, neuronal nuclear protein; CRC, Cajal–Retzius cells; CP, cortical plate; BACE1, β-site APP cleaving enzyme 1; BACE2, β-site APP cleaving enzyme 2; ERG, ETS transcription factor ERG; RUNX1, Runt-related transcription factor 1; DYRKA1, dual-specificity tyrosine-(Y)-phosphorylation-regulated kinase 1A; GATD3A, glutamine amidotransferase like class 1 domain containing 3A; KCNA3, potassium voltage-gated channel; PCNA, proliferating cell nuclear antigen; Ubi-B+1, ubiquitin; SYNJ1, synaptojanin1; RCAN1, regulator of calcineurin; DSCR1, DS critical region gene 1; C21orf5 mRNA, chromosome 21 open reading frame 1; GIRK2, G protein-activated inward rectifier potassium channel 2; WB, Western blotting; IHC, immunohistochemistry; BW, brain weight; CT scan, computed tomography; MRI scan, magnetic resonance imaging; EGL & IGL, external and internal granular layers; SA, surface area; CT, cortical thickness; TBV, total brain volume; GM, grey matter; WM, white matter; STG, superior temporal gyrus; TIV, total intracranial volume; LVA, lateral ventricular area; CSF, cerebrospinal fluid; Wide age range, newborn, neonatal, child & adult; TVUS, transvaginal ultrasound examination; TBSS, tract-based spatial statistics; DTI, diffusion tensor imaging; GSAP, gamma-secretase activating protein; PKNOX1, PBX/knotted 1 homeobox 1; FABP7, fatty acid binding protein 7; NFTs, neurofibrilary tangles; IGP, interlaminar glial palisade; AtD, Alzheimer’s type of dementia; DSAD, DS with Alzheimer’s disease; NS, not significant; IRS1, insulin receptor substrate 1; IR, insulin receptor.

The hippocampus functions as the consolidation of long-term memories from short-term memories (Anand and Dhikav, 2012; Duane and Gregory, 2018b). While there were no differences in the hippocampus in 2–3 years children (Gunbey et al., 2017), in both 7–16 years children and adolescents and 23–51 years adults with DS, the hippocampus was significantly reduced 1.2-folds (Kesslak et al., 1994; Carducci et al., 2013) when compared with controls. Hippocampal area also decreased 1.8-folds in over 40 years old individuals with DS (Pearlson et al., 1998). This suggests that the reduction of the hippocampus starts in childhood and continues throughout life. Hippocampus hypoplasia may interrupt neurogenesis and synaptogenesis, which may affect the functional capacity and causative issues of ID in DS (Kesslak et al., 1994; Pearlson et al., 1998; Carducci et al., 2013) (Figure 1B and Table 1).

### Cerebellum

The cerebellum starts to develop at 6th weeks of gestation, and primitive cerebellar hemispheres appear at 7–9 weeks of gestation after progressive proliferation of the rhombic lip (Sadler, 2012; Duane and Gregory, 2018a). Fissures and lobules of the cerebellum start to form at 10–11 weeks of gestation and continue till birth (Duane and Gregory, 2018a). Cerebellar volume was reduced 1.3-folds in foetuses with DS at 15–38 weeks of gestation (Guihard-Costa et al., 2005) and 1.5 folds in 5–23 year old individuals with DS (Pinter et al., 2001). Around 20 weeks of gestation, white matter (WM) & grey matter (GM) volume in cerebellum reduces 1.4-folds in foetuses with DS (Guidi et al., 2011). GM volume decreases 2.2-folds and WM volume decreases 1.2-folds in 2–3 years old children with DS and significantly decreases in 7–16 years children and adolescents with DS (Carducci et al., 2013; Gunbey et al., 2017). Reduction of grey and white matter volume may cause a decrease in neuron number, relating to neurodevelopmental delay and dementia (Weis et al., 1991; Stoodley, 2016). At 17–21 weeks of gestation in foetuses with DS, the four major fissures (sulci) dividing the cerebellum into five lobes are clearly identifiable near midline of the cerebral vermis in both normal and foetuses with DS, but the fissures gradually become less noticeable in the lateral direction and the lobes become smaller, even invisible (Guidi et al., 2011). The length of the cerebellar fissures, important for dividing the brain into lobes, and the size of the lobules (gyri), are both significantly reduced in foetuses with DS. The fissure length is 1.2-folds smaller, and the lobule size is 1.4-folds smaller (Guidi et al., 2008; Guidi et al., 2011). Fissure length decreases in DS may be responsible for smaller lobes and brain regions (Guidi et al., 2008). These suggest that hypoplasia of the cerebellum in individuals with DS starts in the 2^nd^ trimester of pregnancy and continue through childhood and adulthood, attributing the characteristics of hypotonic and motor coordination abnormalities (Pinter et al., 2001) (Figures 1A,B and Table 1)

### Brainstem

The brain stem develops from the caudal primitive neural tube by the rapid production of specific nuclei of basal and alar plates between the 4th and 12th weeks of gestation (Stiles and Jernigan, 2010; Sadler, 2012). There is significant volumetric reduction of the brainstem (about 1.1 folds), thallamus (1.1-folds) and caudate nucleus (1.14-folds) in children with DS (2–3 years old) (Gunbey et al., 2017). Brainstem volume decreases significantly in 7–16 year old children and adolescents with DS (Carducci et al., 2013) and 1.1-folds in 30–45 year old individuals with DS, but that was not statistically significant (Weis et al., 1991). The area of the pons, midbrain, medulla oblongata, and whole brainstem decreases 1.9-folds, 1.3-folds, 1.1-folds, and 1.4-folds, respectively. in children with DS of 2 days–11 years (Fujii et al., 2017). The brainstem volume reduction may affect the structural connectivity between the brain stem and the cerebrum and cerebellum, which may be responsible for the neurodevelopmental delay in individuals with DS_28_ (Figures 1A,B and Table 1).

### Ventricles

Brain ventricles derive from the cavity of the neural tube at the end of the 3rd week of pregnancy (Figure 1A). The choroid plexus is formed by the tortuous ependymal cells together with vessels and connective tissue and starts to produce cerebrospinal fluid (CSF) at the end of the 1st trimester (Duane and Gregory, 2018a). In the normal foetus in the first trimester of pregnancy, an increase in the size of the lateral cerebral ventricles has been seen at 11–13 weeks of gestation, but its area significantly decreases by 1.9-folds in foetuses with DS (Loureiro et al., 2012). It may be due to abnormal development of the ventricular system and choroid plexus, plus reduction of brain volume. However, at 30 weeks of gestation, lateral and fourth ventricular size increased in foetuses with DS (Baburamani et al., 2019). Similarly, cerebral ventricular hypertrophy has been observed in adults with DS at 30–45 and over 40 years old, with ventricles increased around 1.3 and 3.9-fold (Weis et al., 1991; Pearlson et al., 1998). This may be associated with the decrease in white and grey matter volume and brain volume in individuals with DS (Weis et al., 1991). In addition, alterations in ventricular size may affect cerebrospinal fluid (CSF) formation, which may interrupt the brain’s interstitial fluid homeostasis (Duane and Gregory, 2018a). CSF decreases 1.1-folds in 5–24 years old individuals with DS (Lee et al., 2016) (Figures 1A,B and Table 1).

## Summary and Knowledge Gaps

The ‘Lissencephalic’ or smooth brain surface, brain volume and weight reduction are consistent with the aging process (2nd trimester to 45 years old), related to general cognitive and developmental deficits, and the early onset of dementia and Alzheimer’s disease in later life, respectively, in DS. Hippocampus hypoplasia (starts in childhood and continues throughout life) and hypoplasia of the cerebellum (2nd trimester of pregnancy and continues through childhood and adulthood) may affect the functional capacity and causative issues of ID, and the characteristics of hypotonic and motor coordination abnormalities respectively in DS. The structural connectivity between the brain stem and the cerebrum and cerebellum was derailed due to the brainstem volume reduction, and the reduction of grey and white matter volume may be responsible for the neurodevelopmental delay in individuals with DS**.** However, there has been no study in DS so far on the human embryonic brain development (beginning around the 3rd gestational week) due to a lack of sensitive and diagnostic testing in the embryonic period. Studies were done mostly in the second trimester of pregnancy and in the childhood, adolescent, and adult stages of life. Most specimens were mainly from the cerebrum and cerebellum. Remarkably, there is no information on brain development with DS, such as neural groove, neural tube, or brain vesicles. It is difficult to say whether neuropathology in DS starts at this early stage, which derails the formation of well-organized brain segments (forebrain, midbrain, and hindbrain) and may hamper normal brain development and functioning, causing the neurodevelopmental delay in DS.

## Brain Development at the Cellular Level

**FIGURE 2:**
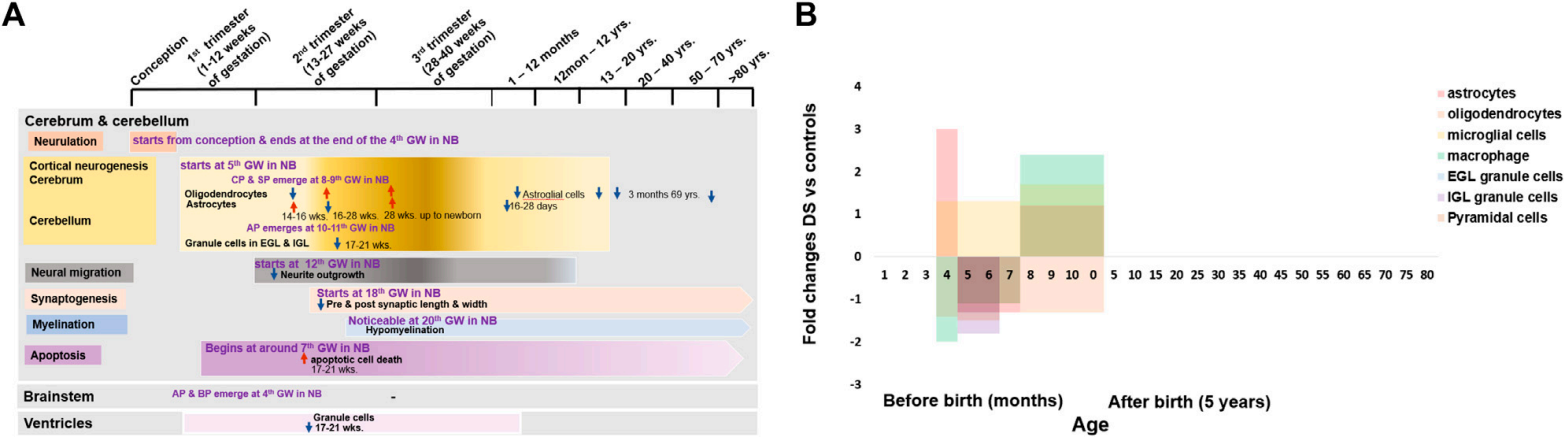
Cellular changes in human Down syndrome brains. **(A)** Time line of major brain cells during development in normal (colour boxes) and Down syndrome (DS, color arrows). **(B)** Fold changes of various brain cell numbers or densities in DS compared with normal controls. Mean change data is extracted from available literature as shown in Table 1. NB = normal brain; DS = Down syndrome; ↑ = increase; ↓ = decrease; GW = gestational week; EGL = external granular layers; IGL = internal granular layers; CP = cortical plate; SP = subplate; AP = alar plate; BP = basal plate.

The infrastructural alterations at the cellular level before birth are a reflection of the modifications in the gross anatomy of the foetal brain (Stiles and Jernigan, 2010). The neural tube is lined by a single layer of pseudostratified epithelium, which proliferates quickly into neuroepithelial cells, collectively named neuroepithelium or the ventricular zone (VZ). All neurons and glial cells (astrocytes, oligodendrocytes, and microglial cells) are derived from the stem cells in the VZ during brain development (Rash and Grove, 2006b; Rhinn et al., 2006; Bystron et al., 2008; Jessell and Sanes, 2000; Noctor et al., 2001). The marginal zone (MZ) and intermediate zone (IZ) show up soon after the development of the VZ from subsequent division of the neuroepithelial cells in the VZ at 4th weeks of the gestation (Stiles, 2008; Tau and Peterson, 2010). These 3 neuroepithelial zones are the basic cellular platform for developing the central nervous system (CNS). The subventricular zone (SVZ) generates the macroglial cells from the fusion of the VZ and IZ (Duane and Gregory, 2018c). Cortical neurogenesis starts from the VZ at 5–6 weeks of gestation, peaks at 15–16 weeks, and ends at the 24th week of gestation (Clancy et al., 2001; Stiles, 2008). Mature neurons develop by the subsequent division of the primary neuroblasts at the 6th week of gestation (Sadler, 2012; Stiles and Jernigan, 2010). After finishing their last cell division at 12–13 weeks, mature neurons start to migrate via radial glia from their origin to destination, which ends at 26–29 weeks of gestation (de Graaf-Peters and Hadders-Algra, 2006; Gupta et al., 2005; Hatten, 1999; Tau and Peterson, 2010). Synapses develop at 18–22 weeks of gestation and throughout life (Tau and Peterson, 2010; Rhinn et al., 2006; de Graaf-Peters and Hadders-Algra, 2006) (Figure 2A). Neuronal apoptosis is a naturally occurring death process of neurons that eradicates cells to maintain the balance of the neuronal cell population. It begins at around the 7th week of gestation and continues throughout life (Blaschke et al., 1996; Rakic and Zecevic, 2000; Lossi and Merighi, 2003; Stiles, 2008) (Figure 2A).

The individual developmental events of the CNS are specific to different regions, which are structurally as well as functionally different from each other (Stiles, 2008; Sadler, 2012). The neural tube modifies its basic cellular platform to accommodate the development of the different brain regions. For the cerebral cortex, the modification is from the emergence of the cortical plate (CP) and the subplate (SP) between the IZ and MZ at 8th–9th weeks of gestation (Bystron et al., 2008). For the cerebellar cortex, modification is due to the presence of alar plate derivatives within the MZ at 10th–11th weeks of gestation. For the brainstem, the posterior and anterior portions of the VZ and IZ modifies into alar cell column or alar plate and basal cell column or basal plate, respectively at the end of 4^th^ weeks of gestation (Duane and Gregory, 2018c; Duane and Gregory, 2018d) (Figure 2A).

### Cerebrum

The cerebral cortex originates from the CP and the SP contains immature neurons that emerge from the VZ. Neuroblasts undergo consecutive proliferation and give rise to mature neurons. Cajal-Retzius cells (CR) arise from the MZ at 5–8 weeks of gestation (Meyer et al., 2000), which regulate the direction and location of the migrating neurons into the appropriate layers of the neocortex (Stiles and Jernigan, 2010). Then mature neurons start to migrate from the proliferative region of the VZ in an orderly manner through the formation of six neuronal layers in the neocortex. The layer-I is the outermost layer near the pial surface that develops from the MZ. The CP generates layer-II to layer-VI, whereas the SP and IZ give rise to subcortical white matter. After reaching their destination in the cortical layers, the neurons start to generate neurotransmitters and neurotrophic factors and widen their processes for communicating with other neuronal cells (Stiles and Jernigan, 2010; Duane and Gregory, 2018c; Duane and Gregory, 2018d). SP acts as a transit for the afferent fibers and premature synapses. They appear in the SP first prior to establishing ultimate connections in the CP (Keunen et al., 2017). The MZ and SP contribute an essential part in the establishment of the cortex but are short-lived brain layers and vanish before birth (Stiles and Jernigan, 2010).

A research study was conducted from 14 weeks of gestation to new-born in the population with DS and a similar-aged normal population on the glial cells of the cerebral cortex and hippocampal region. They identified fewer neuron numbers and disruption in myelination in the cerebral cortex and hippocampal regions in the population with DS (Kanaumi et al., 2013). Astrocytes are increased 3-folds at 14–16 weeks but decreased 1.3-fold at 16–28 weeks, and then again increased 1.2-fold at 28 weeks onward, up to new-born. Oligodendrocytes are decreased 1.4-fold at 14–16 weeks but increased 1.1-fold at 16–28 weeks and 1.3-fold at 28 weeks onward up to new-born, microglia are increased 1.3-fold at 16–28 weeks and 1.7-fold at 28 weeks onward up to new-born, and macrophages are decreased 2-fold at 14–16 weeks and 1.1-fold at 16–28 weeks and increased 2.4-fold at 28 weeks onward up to new-born in DS group. Astrocytes have an important role in neuronal development, durability, and metabolic function. Altered astrocyte production may cause defective neurogenesis and decreased neuron numbers. Disruption of oligodendrocyte production may involve abnormal proliferation and differentiation of oligodendrocyte progenitors, resulting in delayed myelination. Abnormal phagocytic macrophage activity may hamper neurogenesis and apoptosis. All these abnormalities in the early stage of brain development in DS may be the leading cause of ID in individuals with DS (Kanaumi et al., 2013). Interlaminar glial palisade (IGP) is a kind of astroglial cells in supragranular layers that has short unbranched processes (intralaminal astrocytic process) at 20–40 days postnatal period and long branched radial processes (interlaminal astrocytic processes) by the 2nd month of life, which involve in the functional organization of the supragranular layers and related to cortico–cortical pathways. Before 10 days old after birth, IGP has a similar distribution in both DS and normal control, but infants with DS at 16–28 days old have a 1.7-fold shorter branching IGP than normal control. Astroglial cells also decrease from 3 months to 69 years old in individuals with DS. This may suggest that there are alterations of IGP (interlaminal astrocytic processes) in supragranular layers in individuals with DS during early infancy, which may be related to the initial patches (amyloid beta plaques) formation for the development of Alzheimer’s type dementia (AtD) in individuals with DS (Colombo et al., 2002; Colombo et al., 2005) (Figure 2B and Table 1).

### Cerebellum

The development of the cerebellar cortex is somehow different from the development of the cerebral cortex. It is due to the changes in the basic cellular platform of the neural tube to accommodate the development of the cerebellar cortex by the appearance of an external germinal or granular layer in the MZ at 10–11 weeks of gestation (Duane and Gregory, 2018a; Duane and Gregory, 2018c). A huge reduction of the total cell population in the developing cerebellar cortex of foetuses with DS was reported; cerebellar granule cells in the external granular layer were reduced 1.3-folds in 17–21 weeks old foetuses with DS. Whereas in the internal granular layer, the cerebellar granule cell reduction was 1.8-folds (Guidi et al., 2008; Guidi et al., 2011). These findings suggest that there is severe deterioration of granule cell proliferation in the developing cerebellum, which may be associated with cerebellar hypotrophy and cerebellar dysfunction in children and adult individuals with DS, causing dysregulation of motor control and association with Cerebellar Cognitive Affective Syndrome (CCAS), characterized by cognitive deficiency (Schmahmann, 2004) (Figure 2B and Table 1).

### Brainstem

For development of the brainstem, the neural tube alters the anterior and posterior portions of the VZ and IZ into the basal plate and alar plate, respectively, at the end of the 4^th^ week of gestation. The motor cranial nerve nuclei originate from the basal cell column, whereas the sensory-motor nerve nuclei originate from the alar cell column (Duane and Gregory, 2018b). However, there was no study found in DS about the brainstem development at a cellular level in humans (Figure 2B).

### Ventricles

Ventricles of the brain are initially lined by a single layer of the pseudostratified epithelium of the ventricular zone and later by a simple cuboidal epithelium, the ependyma (Duane and Gregory, 2018c). From the 17th-21st gestational weeks, the ventricular zone (of the parahippocampal region that surrounds enclosed the tip of the ventricle) has neurogenesis impairment in foetuses with DS. The total number of granule cells decreased 1.9-folds in the foetuses with DS (Guidi et al., 2008). This suggests that foetuses with DS have fewer proliferating cells in the ventricular zone, which may hamper mature neuron production and migration to the neocortex (Figure 2B and Table 1).

## Summary and Knowledge Gaps

Defective neurogenesis, delayed myelination, and apoptosis are caused by altered astrocytes, oligodendrocytes, microglia, and macrophages during the 2nd trimester of pregnancy and the neonatal period with DS, which are the leading causes of ID in people with DS. Alterations of IGP in supragranular layers during early infancy with DS may be related to the development of early onset dementia in individuals with DS. Deterioration of granule cell proliferation in the developing cerebellum in the 2nd trimester of pregnancy with DS may cause cerebellar hypotrophy and cerebellar dysfunction, which may be responsible for dysregulation of motor control and association with Cerebellar Cognitive Affective Syndrome (CCAS), characterized by cognitive deficiency in children and adult individuals with DS. So far, no cellular study has been found for DS in humans at the early embryonic stage and no study for brainstem development. So, there is a lack of information about DS neuropathogenesis at this critical neurodevelopmental stage. The basic cellular platform plan of the modification of the neural tube for the different regions of brain development in DS is also unknown. Therefore, it is not clear whether there are any alterations at this stage of development in the DS, which may influence the neurogenesis causing ID and cognitive deficiency in children and adult of individuals with DS.

## Brain Development at the Molecular Level

The proliferation and differentiation of the neuroepithelial cells into neurons and glial cells during the early embryonic period is the outcome of complex molecular signaling regulated by multiple gene products (proteins). Molecular signals originate from the neural groove (primitive node) and transmit from one to another consecutively, controlled by the expression of genes in the cells (Stiles, 2008; Stiles and Jernigan, 2010). Deviation of these signals may be attributed to abnormal neural functions in DS during development.

### Genes of Chromosome 21 Related to the Brain With Down Syndrome

**FIGURE 3:**
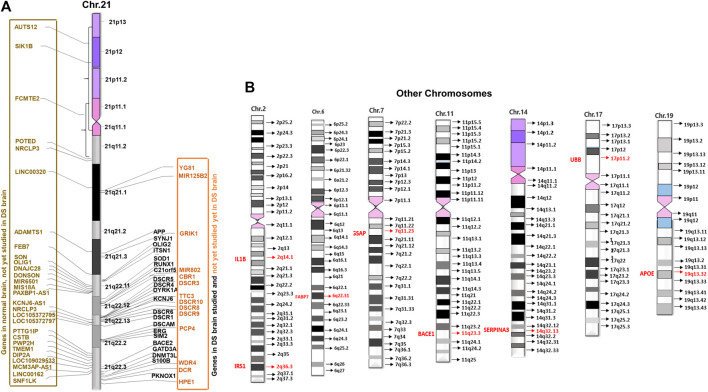
Distribution of brain development genes in human chromosomes. **(A)** Studied and not yet studied brain development genes in normal and brain with DS in Chromosome 21 (Chr21). **(B)** Studied brain development genes in other chromosomes in the brain with DS.

The triplication of chromosome 21 (Chr21) is the culprit of DS. According to the Human Genome Project (HGP), there are 22,000–25,000 genes in the human genome. Chr21 is the 2nd chromosome that has been completely sequenced after Chr22 (International Human Genome Sequencing Consortium, 2001; Venter et al., 2001; International Human Genome Sequencing Consortium, 2004). By that time in 2000, the sequencing of Chr21 disclosed that there were 225 genes and 59 pseudogenes. Among 225 genes, 127 were defined as known genes and 98 as predicted genes (Hattori et al., 2000). So far 75 more genes have been revealed. Currently, there are 202 known genes and 23 predicted genes in the Chr21. Among all, 44 genes are linked to DS disorders, whilst 158 genes are not yet linked to DS disorders according to “GeneCards” (^2^) and ‘NCBI’ (^3^). Out of 44 DS linked genes, 35 genes were associated with DS brain disorders and 9 genes with other DS disorders, whereas out of 158 unlinked genes, 28 genes were associated with brain disorders and 130 genes with other disorders. Surprisingly, only 21 genes (10.40%) of Chr21 were studied in human brains with DS. These studied 21 genes are DYRK1A, S100B, OLIG2, C21orf5/DOPEY2, DSCAM, SYNJ1, ITSN1, GATD3A, SOD1, ERG, APP, BACE2, DSCR1/RCAN1, DSCR5, RIPPLY3/DSCR6, SIM2, DNMT3L, PKNOX1, DSCR4, KNCJ6/GIKR2, and RUNX1. That means not all genes that are linked with brain disorders have been studied in human brains with DS yet (Figure 3A).

### Functional Correlation of Chromosome 21 Genes in the Brain With Down Syndrome

Researchers and scientists continuously make efforts to understand the mechanism of ID in DS in order to identify any potential therapeutic targets to treat the condition. Some research studies have been conducted from several gestational weeks to older adults with DS and compared with age-matched normal controls (Table 1).

**FIGURE 4:**
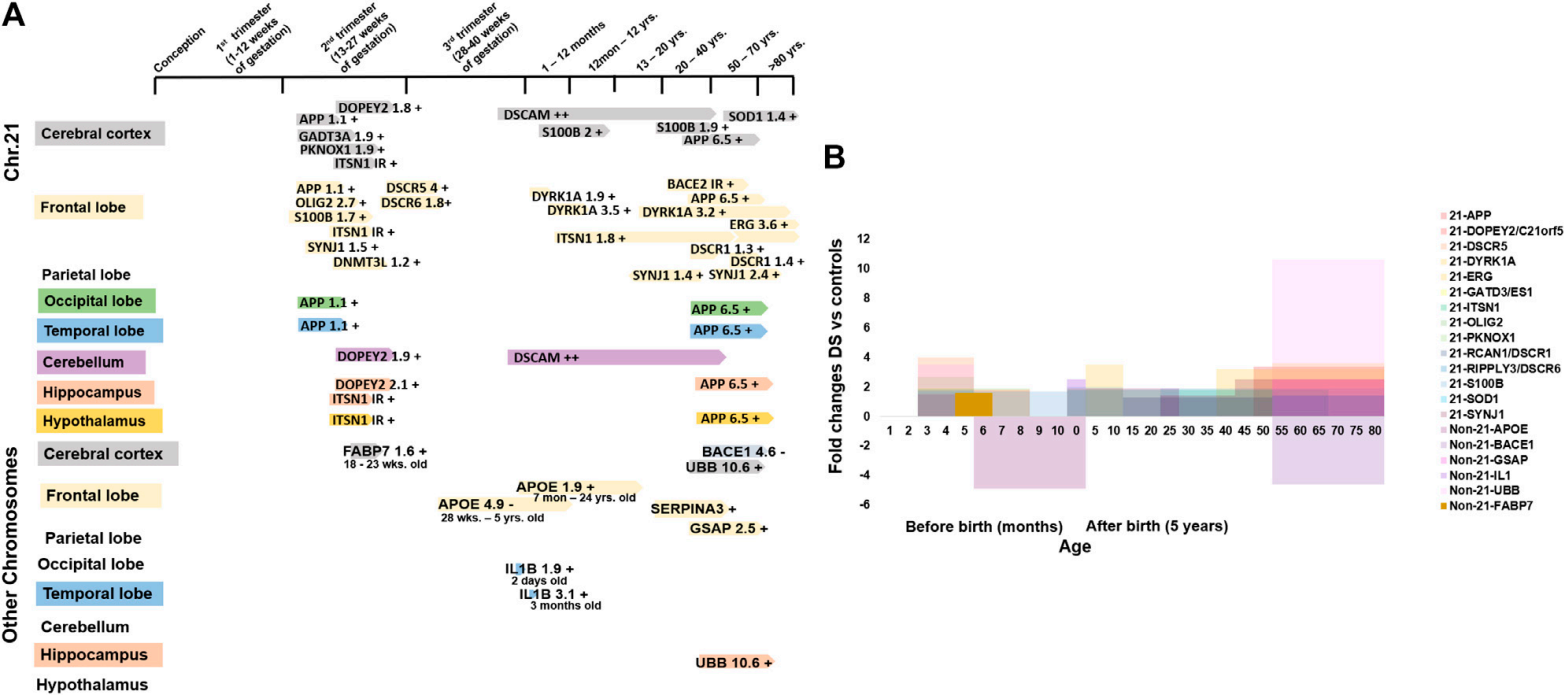
Molecular changes in human Down syndrome brains. **(A)** Time line of studied brain development genes during development in chromosome 21 (Chr21, upper panel) and other chromosomes (non-Chr21, lower panel) in brains with DS. +/- = increase/decrease expression levels compared to normal control brains. **(B)** Fold changes of studied brain development gene expression in Chr21 & non-Chr21 in brains with DS. Mean change data is extracted from available literature as shown in Table 1.

For neurogenesis, DYRK1A is involved in brain growth and development by regulating neurogenesis, neural plasticity, and survival. Phosphorylation regulates the polarization and depolarization of actin filaments as well as aggregation of the DYRK1A-actin complexes (^4^; Guo et al., 2010; Bronicki et al., 2015). DYRK1A expression increased 1.9-folds, 3.5-folds, and 3.2-folds in new-borns, infants, and adults’ frontal cortex with DS on the actin cytoskeleton protein when compared with normal controls. Derangement of DYRK1A-actin aggregation was found in newborns and infants with DS, suggesting that increased DYRK1A expression was related to the abnormal neuronal growth and defective neuronal circuits in brain development by reducing the actin cytoskeleton in the brain with DS, which may be associated with the ID of individuals with DS (Dowjat et al., 2012). S100B is associated with neuron differentiation, neurite extension, astrocytosis, and axonal proliferation (Allore et al., 1990; ^5^). S100β^+^ astrocytes increased 1.7-fold at 17–35 weeks of gestation, 2-fold at 8 months to 9 years old and 1.9-fold at 22–68 years old with DS. The S100β^+^ astrocytes increase the intraneuronal free calcium level and stimulate the formation of abnormally prominent and tortuous processes in the neurons of DS, which may be the cause of the ID in DS (Griffin et al., 1998). OLIG2 is essential for the development of oligodendrocyte and somatic motor neurons in the hindbrain and spinal cord (Ono et al., 2008). Potassium channel KCNA3 is involved in the oligo-progenitor differentiation and proliferation changes (Prüss et al., 2011; Zhou et al., 2011) and OLIG2 controls the KCNA3 channel expression level (Lu et al., 2012). Overexpression of the OLIG2 (2.7-folds) diminishes the potassium channel (1.7-folds) activity, causing a reduction of oligodendrocyte progenitor proliferation in foetuses with DS (14–18 weeks), which may be the reason for smaller hindbrain size, developmental delay, and ID in DS by inhibiting neuronal proliferation and resulting in neuronal reduction and hypomyelination (Lu et al., 2012) (Figures 4A,B and Table 1).

For neuronal growth, C21orf5/DOPEY2 is involved in selective transcriptional activity and regional and cellular specificity of gene transcripts in early development (Rachidi et al., 2000). The differential expression of C21orf5 is restricted to the foetal cerebral (lower intensity), hippocampal (lower intensity) and cerebellar (highest intensity) regions, which are associated with learning and memory processes through neuronal proliferation and distribution, dendritic arborization and myelination (Rachidi et al., 2006). In the foetal (22–24 week) cerebral cortex with DS, cerebellum, and hippocampus, C21orf5 is differentially overexpressed by 1.8-folds, 1.9-folds, and 2.1-folds, respectively (Rachidi et al., 2009). C21orf5 overexpression in those regions of the developing brain with DS may suggest a potential role in the mechanism of ID in DS by alteration of dendritic densities, retardation, and disorganization of cortical lamination and slow myelination. DSCAM has a role in nervous system development, including axon guidance and segregation, dendritic patterning, and synapse formation (Jianhua et al., 2011). DSCAM was detected in both the brain with DS and the normal brain at 40 GW in the cerebral and cerebellar white matter, expression level was increased thereafter with advancing age. But it was increased more during childhood and adulthood of brains with DS. DSCAM was also detected in the dystrophic neurites of the senile plaques in the cerebral cortex of adults with DS (Saito et al., 2000; Amano et al., 2008). This suggests that DSCAM overexpression may be involved in abnormal neuronal projections and axonal plasma membrane (myelin-sheath) formation, causing delayed myelination and disruption of neural circuit that may be associated with the development of the ID in DS, and may also take part in the early onset of Alzheimer’s disease by disrupting axonogenesis and synaptogenesis, and senile plaque formation (Saito et al., 2000; Huang et al., 2011). SYNJ1 and ITSN1 play a role in clathrin-mediated synaptic vesicle endocytosis, recycling, and transmission and perhaps may be associated with cell signaling (Lee et al., 2004; ^6^; Tsyba et al., 2004; O'Bryan, 2010). Synaptojanin increased 1.5-fold in 15–16 weeks old foetuses with DS than in control foetuses during the peak period of neuronal migration (Arai et al., 2002). ITSN1 expression level increased in foetal (18–22 weeks) brains with DS (Pucharcós, 1999). It was increased by 1.8-folds in DS (5 months–62 years) when compared with controls (6 months–67 years) frontal cortex (Michael et al., 2011). Synaptojanin and ITSN1 overactivity, associated with neuronal loss, atrophic basilar dendrites, and atypical synaptic density, disrupt neuronal migration and synaptic transmission in the developing brain with DS by defective endocytic processes, elevating Ras activation and increasing neuronal receptor trafficking and neurodegeneration (Pucharcós, 1999; Arai et al., 2002), which may disrupt normal brain functioning and cause the development of ID in DS. SYNJ1 also increased 1.4-folds in individuals with DS vs. young control, 2.5-folds in DS developed AD (DSAD) vs. old control, and 4.9-folds in DSAD vs. AD patients (Martin et al., 2014). The overall high SYNJ1 levels in DS may be related to abnormal synaptic vesicle formation or synaptic loss, leading to neurodegeneration in patients with DS (Figures 4A,B and Table 1).

For neuron damage, GATD3A/ES1 encodes a mitochondrial protein, which is a member of the DJ-1/Pfpl gene family relating to basic mitochondrial functioning (^7^). GATD3A was overexpressed by 1.9-folds in the foetal brain with DS when compared with control (Shin et al., 2004). This result suggests that overexpression of GATD3A may be associated with the ROS related neuron apoptosis and neurodegeneration in the foetuses with DS, causing ID in DS (Sinet, 1982; Busciglio and Yankner, 1995). SOD1 detoxifies the free toxic superoxide radicals to molecular oxygen and hydrogen peroxide and protects the neurons from oxidative injury (Pardo et al., 1995; Niwa et al., 2007). Overexpression of SOD1 was seen in cerebral cortex with DS (over 50 years) by 1.4-folds (Gulesserian et al., 2001a,) whereas in foetal brains with DS it was not overexpressed (Gulesserian et al., 2001b). This higher level of SOD1 may give rise to an abnormally high hydrogen peroxide level in the neurons. This could result in free radical damage to the nerve cell membrane and may relate to ID in DS (Brooksbank and Balázs, 1983). ERG may be involved in transcriptional regulation of genes important for embryonic development, cell proliferation and differentiation, angiogenesis, and apoptosis (Yi et al., 1997; Trojanowska, 2000; Oram et al., 2010). ERG was overexpressed by 3.6-folds in cerebral cortex with DS (>50 years) (Shim et al., 2003). Overactivity may be related to apoptotic neuronal death and neurodegeneration, causing cognitive failure and dementia in DS. APP also plays a role in neuronal and axonal growth (Adler et al., 1991). BACE2 encodes a glycoprotein that splits APP into AβP, which promotes the amyloid plaque formation in AD and the brain with DS causing dementia (Solans et al., 2000; Ehehalt et al., 2002). APP is overexpressed by 3.4-fold in the adult (>50 years) brain with DS while it is comparable in the fetal (18–19 weeks) brain with DS (Cheon et al., 2008). BACE2 is present in the neurons of the neurofibrillary tangles of brains with DS (49–60 years) with dementia of the Alzheimer’s type (DAT), but not in those without DAT (27 gestational week (GW) to 32 years) (Motonaga et al., 2002). This suggests that in the fetal stage, APP and BACE2 were not exacerbated, and overexpression of APP and BACE2 in adults with DS promotes accumulation of AβP and participates in neurodegeneration (Oyama et al., 1994; Cheon et al., 2008) (Figures 4A,B and Table 1).

A region of the long arm of Chr21 (21q22) containing most of the genes, known as the Down syndrome critical region (DSCR), is supposedly responsible for the development of the DS phenotypes (Nakamura et al., 1997; Shapiro, 1999). DSCR1/RCAN1 inhibits the calcineurin dependent signaling pathway by interacting with calcineurin A and may play a role in CNS development (Fuentes et al., 1995; Juan et al., 2000). RCAN1 was overexpressed by 1.3-folds (over 23 years) and 1.4-folds (over 50 years) in the adult brain with DS than in controls, and this chronic elevation of expression may be involved in the formation of NFTs (neurofibrilary tangles) in DS, like in AD (Ermak et al., 2001; Perluigi et al., 2014). The DSCR4 gene is involved in the development of DS (Nakamura et al., 1997). The gene encodes an enzyme that activates the first reaction of glycosylphosphatidylinositol (GPI) biosynthesis (Watanabe, 2000). DSCR6/RIPPLY3 gene functions as a transcriptional repressor and takes part in the development of pharyngeal apparatus and derivatives (Kawamura et al., 2005). The expression levels of DSCR5 and DSCR6 were increased by 4-folds and 1.8-folds in foetuses with DS when compared with the normal controls. No immunoreactive band was observed for DSCR4 in the fetal cortex with DS. This suggests that all gene products from the DSCR are up-regulated as anticipated by the gene dosage hypothesis (Ferrando-Miguel et al., 2004). The KCNJ6/GIRK2 gene modulates the circuit activity in the neuronal cell and the heart rate in cardiac cells through G-protein coupled receptor stimulation (^8^). KCNJ6 was decreased by 2.5-folds, but the result was not significant in foetuses with DS when compared with the normal controls (Ferrando-Miguel et al., 2004). RUNX1 regulates the transcription of target genes and is important for the development of normal hematopoiesis (Cohen Jr, 2009). In the frontal cortex with DS, RUNX1 was overexpressed by 1.3-folds when compared with age-matched (over 50 years) controls, but the result was not statistically significant (Shim et al., 2003) (Figures 4A,B and Table 1).

### Genes of Non-Chr21 Related to the Brain With Down Syndrome

Research studies have found the involvement of some non-Chr21 genes in neuropathogenesis in DS through trans-acting reactions. There were 13,695 disease genes recorded in “GeneCards” (^9^) and “NCBI” (^10^). Currently, only 16 non-Chr21 genes have been linked to DS in “GeneCards (^9^) and NCBI” (^10^). Among those, 12 genes were studied in humans with DS disorders: 8 genes for brain disorders with DS 2 for heart disorders with DS, and 2 for leukemia with DS. These 8 genes are APOE (19q13.32), BACE1 (11q23.3), FABP7 (6q22-23), IL1B (2q14.1), GSAP (7q11.23), SERPINA3 (14q32.13), UBB (17p11.2), and IRS1 (2q36.3) (Figure 3B).

### Functional Correlation of Non-Chr21 Genes in the Brain With Down Syndrome

For neuron damage, IL1B/IL1 encodes a protein of interleukin 1 cytokine family member origin from activated macrophages (March et al., 1985; Vidal-Vanaclocha et al., 2000). IL1 is generated by glial cells and stimulates gliosis (Fontana et al., 1982; Giulian et al., 1988). In the temporal lobe, IL1 immunoreactive cells increased 1.9-folds and 3.01-folds in 2 days old and 3 months old with DS when compared with neonates without DS. This suggests that IL1B overexpression escalated the proliferation and activity of the astroglial cell and may be associated with the ID of DS by astrogliosis (Griffin et al., 1989) (Figures 4A,B and Table 1).

For AD, APOE is involved in lipoprotein-mediated lipid transport into the organs, especially in the brain (Blum, 2016; Chappell, 1989). APOE, generated by the astrocytes, has an essential role in the formulation and redistribution of lipids for growth and repair at the time of neuro-development and injury. Apo-E is also one of the elements of amyloid deposition. Apo-E in astrocytes is decreased by 4.9 folds in the white matter of 28 GW to 5 year old individuals with DS and increased by 1.9 folds in the frontal cortex of 7 months–24 year old individuals with DS. This defective and altered production of apo-E-producing astrocytes in the developing stage may point to the early onset of dementia in individuals with DS (Arai et al., 1995). GASP activates the Aβ formation (He et al., 2010), whose expression level is elevated 2.5-folds in the adult frontal cortex with DS, and this overexpression may suggest the accumulation of increased Aβ resulting in AD pathology in DS (Chu et al., 2016). UBB encodes ubiquitin, which is involved in regulating gene expression, the maintenance of chromatin structure and the stress response (Baker and Board, 1987). Ubi-B^+1^ is increased by 10.6-folds in the cerebral cortex with DS. No βAPP^+1^ was observed in the control brain, whereas, in the brain with DS, βAPP^+1^ was observed to be 86% increased (van Leeuwen et al., 1998). Overexpression of the UBB causes excessive production of Ubi-B protein and may relate to altered neuronal functioning in DS. Excessive βAPP^+1^ and Ubi-B^+1^ in the neurofibrillary tangles and dystrophic neurites may suggest the relationship between the two proteins and their involvement in causing AD in individuals with DS (van Leeuwen et al., 1998) (Figures 4A,B and Table 1). IRS1 encodes a protein which is phosphorylated by insulin receptor (IR) tyrosine kinase and mediates the control of various cellular processes by insulin. IRS1 & IR proteins level decreased 1.43-folds & 2-folds, respectively, in the frontal cortex of <40 years old individuals with DS when compared with similar aged controls although the results were not statistically significant. This may interfere with the insulin signaling pathway and impair glucose uptake in the brain with DS, causing the brain to become insulin resistant and hamper the physiological functions of insulin, such as neurite growth, promoting dendritic spine formation, development of excitatory synapses, and promoting neuronal survival by inhibiting apoptosis; leading to the development of neuropathology like AD in young individuals with DS (Ogihara et al., 1997; Tramutola et al., 2020) (Figure 3B and Table 1).

### Molecular Interaction Between Chromosome 21 and Non-Chr21 Genes

**FIGURE 5:**
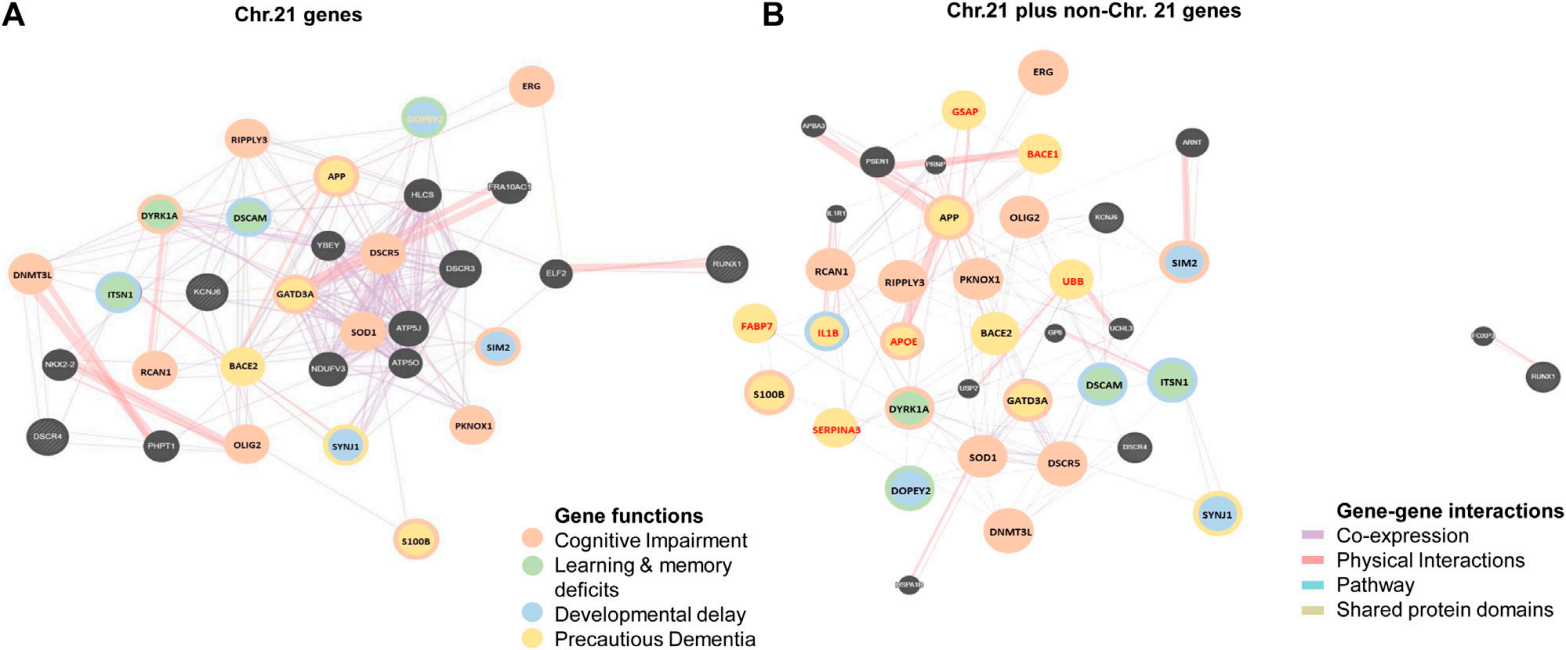
Molecular networks in human Down syndrome brains. **(A)** Network of studied brain development genes in chromosome 21 (Chr21) in brains with DS. **(B)** Network of studied brain development genes in Chr21 (gene names in black) together with non-chromosome 21 (non-Chr21, gene names in red) in brains with DS Different color circles and lines represent the groups of genes with similar gene functions and interactions in metabolic pathways, physical interactions, gene co-expression or sharing their protein domains.

**FIGURE 6:**
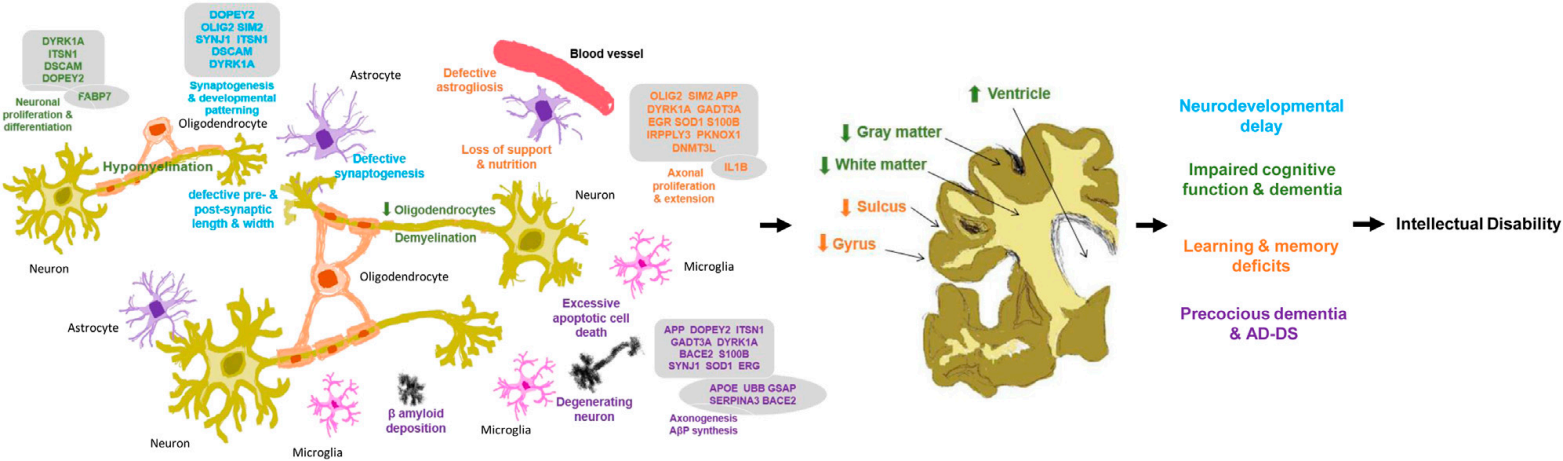
Neuropathology in Down syndrome brain in human. Relationship of brain development genes, molecular, cellular, anatomical changes and clinical manifestations for the development of intellectual disability in Down syndrome. Genes in boxes as chromosome 21 genes; genes in circle as non-chromosome 21 genes. Changes in blue lead to defective synaptogenesis and pre- and post-synaptic length and width, thereafter neurodevelopmental delay; green lead to reduced oligodendrocytes and hypo- and de-myelination, thereafter impaired cognitive function and dementia; orange lead to defective astrogilosis and loss of support and nutrition thereafter learning and memory deficits; and purple lead to degenerating neuron, excessive apoptosis and β-amyloid deposition, thereafter precocious dementia & AD-DS. Arrows ↑/↓ as direction of the gene expression and changes.

Based on the molecular network built amongst the genes of Chr21 (Figure 5A), groups of genes similar in function are linked together either by metabolic pathways, physical interactions, gene co-expression or by sharing their protein domains involved in the DS phenotypic abnormalities. DYRK1A, DOPEY2, ITSN1, and DSCAM regulate neuronal proliferation and differentiation through the cell signaling pathway. Overexpression of these groups of genes may be responsible for learning and memory deficits. PKNOX1 and DNMT3L activate the transcription factors and epigenetic changes. The DNMT3L gene is essential for the establishment of maternal genomic imprints (Ooi et al., 2007) and plays a potential role in DS neurodevelopment through gene expression modulation in neurons (Feng et al., 2010). The methylation profiles in the neuro progenitors showed significant global hypermethylation in the fetal (18 week) frontal cortex with DS. DNMT3L may play a role in the epigenetic changes of other genes in DS (Lu et al., 2016). OLIG2, SIM2, S100B, SOD1, DYRK1A, APP, and GADT3A are involved in axonal proliferation and neurite extension, and overactivity of this group of genes may be responsible for cognitive impairment. The SIM2 gene encodes a transcription factor regulating midline cell fate determination, which is a master regulator of neurogenesis (Dahmane et al., 1995; ^11^). Differential expressions of SIM2 in the cerebral cortex (weak), hippocampal formation, and cerebellar cortex (strong) in the embryonic and foetal brain suggest the potential roles in the development of the CNS (Rachidi et al., 2005). In the frontal cortex with DS, SIM2 was overexpressed by 1.3-folds when compared with age-matched (over 50 years) controls, but the result was not statistically significant (Shim et al., 2003). Based on the networks, SIM2 seems to be a master gene in regulating other associated genes during brain development. Disruption of SIM2 may contribute to neuropathogenesis in DS in early life (Figures 4B, 5A, 6 and Table 1).

DOPEY2, SYNJ1, SIM2, DSCAM, DYRK1A, ITSN1, and OLIG2 are involved in synaptogenesis, dendritic arborization, and developmental patterning. Overactivity of this group of genes may contribute to developmental delay. APP, BACE2, S100B, GATD3A, DOPEY2, DYRK1A, ITSN1, and SYNJ1 are involved in axonogenesis, AβP synthesis, neuronal adhesion, and neurite growth, and overexpression of this group of genes may be responsible for precautious dementia and AD-DS. The βAPP^+^ neurons were increased 5-folds in 8 months–9 years old and 12-folds at 22–68 years old individuals with DS, which has a significant correlation with the S100β^+^ astrocyte numbers in DS when compared with the controls. This suggests that overexpression of the S100B gene may interact with APP and participate in the formation of amyloid plaques at the early age of individuals with DS, causing Alzheimer’s like dementia (Griffin et al., 1998) (Figures 4B, 5A, 6 and Table 1).

While non-Chr21 genes are included in the network, non-Chr21 genes disperse the Chr21 genes placement in the network, which may be due to gene interactions, changing metabolic pathway, and transactivation of gene expression (Figure 5B). The interactions of Chr21 genes become dissociative within the network and may suggest disturbance between gene molecules in the neural manifestations. Non-Chr21 genes have strong interactions with Chr21 genes, mostly interacting with those, involved in AD like dementia in DS. For example, APOE (non-Chr21), DSCAM, and S100B (Chr21) are involved in axonal proliferation, lipid redistribution, and developmental patterning, and their overexpression may be responsible for cognitive dysfunction. IL1B (non-Chr21) and S100B (Chr21) are involved in astroglial cell proliferation, and activation and interactions may be responsible for abnormally prominent and tortuous neural process formation, causing ID in DS (Griffin et al., 1989; Griffin et al., 1998) (Figures 4B, 5B, 6 and Table 1).

UBB (non-Chr21) and DYRK1A, DSCR5, PKNOX1, DSCR4, OLIG2, RIPPLY3, DOPEY2 (Chr21) are involved in maintaining the chromatin structure and regulating stress response, and their interactions may be responsible for developmental delay (Saito et al., 2000; Amano et al., 2008; Lu et al., 2012). PKNOX1 activates homeodomain transcription factors that are involved in embryonic development and organogenesis (Berthelsen et al., 1998; Ferretti et al., 2006). PKNOX1 and FABP7 were overexpressed by 1.9 and 1.6-folds in the foetal (18–23 weeks) brain with DS. FABP7 plays an important role in radial glial fiber formation that is essential for the migration of immature neurons to create cortical layers during brain development (Anthony et al., 2005). Overexpression of FABP7 may result in the functional disturbance of glial cells, hampering neuronal migration and causing neurodegeneration. Luciferase expression increased 3.5-folds through the FABP7 promoter with PKNOX1, and the expression increased 1.4-folds by deleting the FABP7 promoter with PKNOX1. This suggests that the transactivation of the FABP7 (6q22-23) gene promoter is due to PKNOX1 (21q.22.3) overexpression and may be indirectly related to DS neuropathogenesis by altering the expression of other genes (Sánchez-Font et al., 2003). APOE, GSAP, BACE1, IL1B, UBB, and SERPINA3 (non-Chr21) interacts with APP (Chr21); and GSAP, BACE1, UBB, and SERPINA3 (non-Chr21) interacts with BACE2 (Chr21). These genes are involved in lipid formulation and redistribution and AβP production, overactivity of these groups of genes may be responsible for AtD in DS. BACE1 activates the first step in AβP formation from APP (Vassar et al., 1999). BACE1 activity increased 2.3-folds in the cerebral cortex of foetuses with DS (18–19 weeks) and was comparable in over 50 years old brain with DS (Cheon et al., 2008). The significant overexpression of BACE1 in the foetus with DS and diminished activity in adults with DS suggests that DS phenotypes are not only due to the gene dosage hypothesis but may also reflect the associated neurodegeneration in the pathogenesis of AD in DS. SERPINA3/ACT inhibits the plasma protease. This protein targets and influences tissue-specific protease like endopeptidases, such as trypsin and chymotrypsin (Chandra et al., 1983; Eriksson et al., 1986). All amyloid plaques and small amyloid deposits of Amyloid beta-protein (AβP) in Senile Dementia of Alzheimer’s Type (SDAT) brain from (>50 years old) DS, AD and normal control had been alpha 1-antichymotrypsin (ACT) positive. These results may suggest that there is an association between ACT and AβP. ACT may be involved in Alzheimer’s type of dementia in DS (Shoji et al., 1991) (Figures 4B, 5B, 6 and Table 1).

## Summary and Knowledge Gaps

Out of 202 genes on chromosome 21, only 21 genes were studied in the human brain with DS and 8 non-Chr21 genes were also studied in the human brain with DS. Not only the APP gene is responsible for dementia in DS, GATD3A, SOD1, ERG, BACE2, DSCR1/RCAN1, APOE (19q13.32), BACE1 (11q23.3), IL1B (2q14.1), GSAP (7q11.23), UBB (17p11.2), and IRS1 (2q36.3) genes may also play a major role in dementia in DS. Whilst DYRK1A, S100B, OLIG2 genes involved in neurogenesis and C21orf5/DOPEY2, DSCAM, SYNJ1, ITSN1 genes help to neuronal growth, targeting these genes in future has great potential for DS treatment. However, no gene studies were found for the brain with DS in humans during the embryonic period (3rd–8th week), which is the origin of the neural groove (primitive node) and the starting point for complex molecular signaling. Vaguely, we are unable to know about the expression of genes in the cells of the neocortical proliferative zone, which are essential for the initial patterning of the neocortex into cortical areas. It may be due to the unavailability of embryo samples and the limitation of current advanced prenatal diagnosis to detect DS in very early gestation. The most accurate screening test, the NIPT (Non-Invasive Pregnancy Test), is only available at 9–10 weeks of gestation. There is no other prenatal test earlier than that gestational age. Since DS starts with conception, we need to know the developmental changes at the very beginning at the molecular level. A mouse model of DS would be the only tool for early brain developmental studies.

## Potential Treatment and Future Prospects

There is no prevention or cure for DS as it is due to non-disjunction during meiosis before conception. Recent modern technology and ongoing experimental studies on DS provide hope for mothers who decide to continue pregnancy after the diagnosis of DS. Since neuropathology of DS starts in the embryonic period and neurogenesis only ceases at birth, prenatal therapy may improve the outcomes of DS neuropathogenesis and restore brain functions at anatomical, cellular, and molecular levels during the fetal and neonatal periods. Preclinical DS studies showed that ID and brain defects can be altered by early pharmacological intervention during pregnancy. For example, dietary choline supplementation during pregnancy and lactation enhanced cognitive functioning and emotion regulation in the Ts65Dn mouse model (Moon et al., 2010). Choline is an important nutrient for the developing brain as it is essential for acetylcholine (Ach) biosynthesis. Ach is the main neurotransmitter in the regulation of neuronal proliferation, differentiation, maturation, plasticity, survival, migration, and synapse formation (Barbara et al., 2016). Dietary choline supplement could be given for all pregnant women to maintain proper health as well as brain development at cellular level and can be a potential early fetal intervention for known and unknown pregnancy with DS (Shoji et al., 1991; Lauder and Schambra, 1999; Jian et al., 2012). In addition, epigallocatechin gallate (EGCG) is a natural polyphenol family member and plays a crucial role in early brain development at the molecular level by inhibiting DYRK1A in transgenic overexpressed Dyrk1A mice during the prenatal period (Guedj et al., 2009; Samantha et al., 2016). Similarly, ALGERNON (as Altered Generation of Neurons) is a kinase inhibitor that promotes neural stem cell (NSC) proliferation by inhibiting DYRK1A in a mouse model of DS (Nakano-Kobayashi et al., 2017).Restoring neurogenesis by inhibiting overexpressed Dyrk1A increases brain growth/volume at anatomical level (Park and Chung, 2013). There is evidence that supplementation of maternal folic acid and multivitamins before and during pregnancy is positively correlated with a reduced risk of ASD with ID in offspring when compared to no supplementation (DeVilbiss et al., 2017; Levine et al., 2018). Researchers continuously make efforts to find new ways to rescue DS neuropathogenesis by early prenatal and postnatal intervention, and hopefully, those therapies can be used in DS prenatal and postnatal intervention. There is an idea for repairing the genetic defects causing DS by the preliminary application of “chromosome therapy,” which unlike gene therapy, which is only for the disease caused by a single gene. Since DS is due to an extra copy of Chr21, “chromosomal therapy” could be applicable to “silence” or turn off the third copy of Chr21 by inserting XIST into Chr21 as in DS pluripotent stem cells (Li et al., 2012; Jiang et al., 2013). We look forward to further development and studies of rescued neuropathogenesis in feotuses with DS and in individuals with DS in the near future.

## Conclusion

The mysterious cause behind ID in DS is yet unknown. It may be considered as an altered anatomical structure, such as decreased brain volume; cellular functions, such as hypocellularity in granular cell layers; and molecular pathways, such as transactivation resulting in dysfunction of higher brain functions. From this review, we came to know that the most crucial time of brain development is the embryonic period, when the main infrastructural changes determine the development of different brain regions. However, for the embryonic brain development in DS, no research information is yet available. So, research interest should be focused on the embryonic period (3rd to 8th weeks) by experimentation of the DS stem cell (e.g., iPS) and mouse models of DS to identify defects, if any.

## Footnotes

1 Early Intervention–NDSS. Ndss.org. https://www.ndss.org/resources/early-intervention/.

2
https://www.genecards.org/cgi-bin/listdiseasecards.pl (12–2018).

3
https://www.ncbi.nlm.nih.gov/gene/(12–2018).

4 NCBI <https://www.ncbi.nlm.nih.gov/gene/1859>

5 NCBI <https://www.ncbi.nlm.nih.gov/gene/6285>

6 NCBI <https://www.ncbi.nlm.nih.gov/gene/8867>

7 NCBI <https://www.ncbi.nlm.nih.gov/gene/8209>

8 NCBI <https://www.ncbi.nlm.nih.gov/gene/3763>

9
https://www.genecards.org/cgi-bin/listdiseasecards.pl (04–2019).

10
https://www.ncbi.nlm.nih.gov/gene/(04–2019).

11 NCBI <https://www.ncbi.nlm.nih.gov/gene/6493>
